# Lipid metabolism and rheumatoid arthritis

**DOI:** 10.3389/fimmu.2023.1190607

**Published:** 2023-05-31

**Authors:** Qian Lei, Jie Yang, Li Li, Ning Zhao, Cheng Lu, Aiping Lu, Xiaojuan He

**Affiliations:** ^1^ Institute of Basic Research in Clinical Medicine, China Academy of Chinese Medical Sciences, Beijing, China; ^2^ Law Sau Fai Institute for Advancing Translational Medicine in Bone and Joint Diseases, School of Chinese Medicine, Hong Kong Baptist University, Hong Kong, Hong Kong SAR, China; ^3^ Shanghai GuangHua Hospital of Integrated Traditional Chinese and Western Medicine, Institute of Arthritis Research, Shanghai Academy of Chinese Medical Sciences, Shanghai, China; ^4^ Guangdong-Hong Kong-Macau Joint Lab on Chinese Medicine and Immune Disease Research, Guangzhou, China

**Keywords:** rheumatoid arthritis, lipid metabolism, inflammation, joint destruction, treatment

## Abstract

As a chronic progressive autoimmune disease, rheumatoid arthritis (RA) is characterized by mainly damaging the synovium of peripheral joints and causing joint destruction and early disability. RA is also associated with a high incidence rate and mortality of cardiovascular disease. Recently, the relationship between lipid metabolism and RA has gradually attracted attention. Plasma lipid changes in RA patients are often detected in clinical tests, the systemic inflammatory status and drug treatment of RA patients can interact with the metabolic level of the body. With the development of lipid metabolomics, the changes of lipid small molecules and potential metabolic pathways have been gradually discovered, which makes the lipid metabolism of RA patients or the systemic changes of lipid metabolism after treatment more and more comprehensive. This article reviews the lipid level of RA patients, as well as the relationship between inflammation, joint destruction, cardiovascular disease, and lipid level. In addition, this review describes the effect of anti-rheumatic drugs or dietary intervention on the lipid profile of RA patients to better understand RA.

## 1 Introduction

Rheumatoid arthritis (RA) is a chronic inflammatory autoimmune disease, which is prevalent in about 0.5–1% of the population. The pathological basis of RA is synovial inflammation, which then erodes the articular cartilage and bone tissue from the synovium, resulting in the destruction of joints and affecting the normal joint function of patients (1–3). Extraarticular manifestations, such as cardiovascular diseases (CVD) and comorbidities are common manifestations of RA, which increase the incidence rate and mortality of RA patients. Although the exact etiology is still unclear, it is currently believed that genetic, autoimmunity, environmental, and intestinal microorganisms are important causes of the disease (4, 5). In addition to these generally acknowledged factors, recent studies suggest that there is also a profound relationship between lipid metabolism and RA.

**Figure 1:**
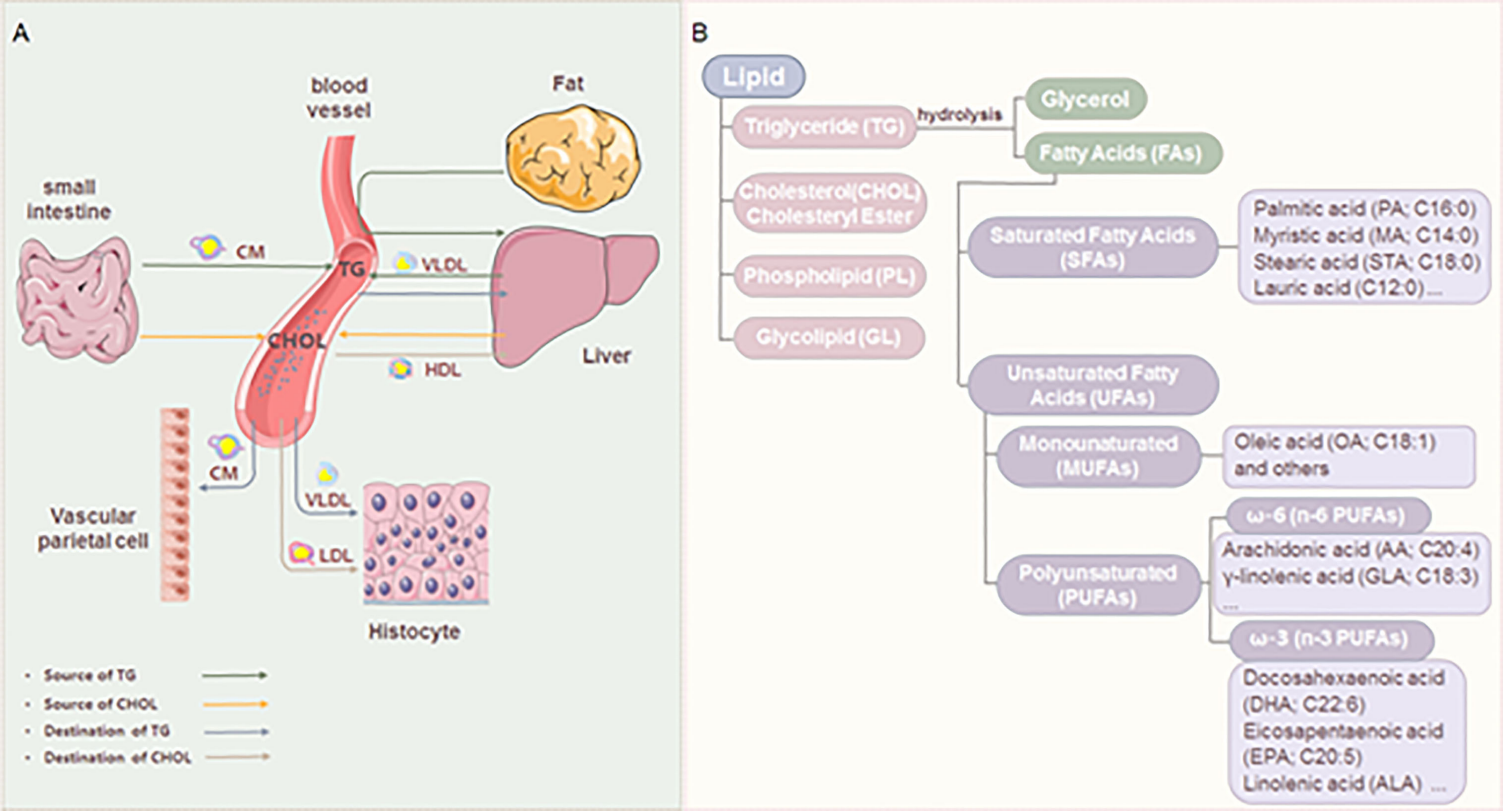
Lipid metabolism and classification. **(A)** The source and destination of triglyceride and cholesterol. Triglyceride (TG): The fat in the food is digested to form chylomicron (8) in the small intestine. TG is carried by CM through blood circulation to adipose tissue for storage. A part of TG in adipose tissue is decomposed into glycerol and FAs, which are transported to the liver. The liver reconstitutes them into TG for storage and can also transport blood in the form of VLDL. Cholesterol (CHOL): absorbed by food, and synthesized by the liver or small intestine; It is carried by LDL and transported to the whole body to synthesize lipid hormones and form cell membranes. The excess CHOL in the tissue is absorbed by HDL, transported to the liver, and then discharged from the body. **(B)** Classification of lipids and FAs.

As the most abundant type of cellular metabolites, lipids are important for energy supply and storage, cell membrane construction, and signal transduction (6). Lipid metabolism is a process of digestion, absorption, decomposition, and metabolism of lipids, such as triglyceride (TG), cholesterol, and fatty acids (FAs) (7), which is an important and complex biological process (**Figure 1**). Under normal conditions, the cholesterol that the body absorbs from food and synthesizes in the liver will be converted into steroid hormones or become a component of the cell membrane, keeping the concentration of cholesterol in the blood constant. The fat in food is absorbed and synthesized into TG through the intestine, stored in the liver or fat, or the blood in a free form. TG is decomposed into glycerol and FAs under the catalysis of enzymes. FAs usually exist in the blood in free form or the body as the main component of some high-order lipids (such as phospholipids and glycolipids). Lipid reprogramming within cells further promotes membrane remodeling, affecting membrane fluidity, lipid raft formation, and proinflammatory signaling cascades (9). In addition, lipids are also the precursors of various molecules with important biological roles. For example, polyunsaturated fatty acids (PUFA) generate oxidized lipids through the action of specific enzymes. Among them, arachidonic acid (AA) in n-6 PUFA and its derivatives, such as prostaglandin (PG), thromboxane, and leukotriene, are mainly considered substrates of pro-inflammatory mediators. Eicosapentaenoic acid (EPA), docosahexaenoic acid (10) in n-3 PUFA, and their derivatives are considered substrates of mediators inducing inflammation regression (11). Lipoxins from AA, E-series resolvins from EPA, D-series resolvins, protectins, and maresins from DHA are called specialized pro-resolving mediators (SPMs), which play an important role in the regression of inflammation (12).

Under normal circumstances, the balance of lipids is maintained by strictly controlling the levels of cholesterol and FAs. But the imbalance of these regulatory mechanisms becomes the foundation of human diseases. Recent studies have shown that RA patients have abnormal lipid metabolism, and these abnormal lipid metabolites affect the progress of RA (13, 14). During the treatment of RA, the lipid level of RA patients is regulated under the effect of drugs. In addition, dietary intervention of lipid intake can also play a role in regulating the lipid level of RA patients. Combined with the complex process of lipid metabolism and the disease characteristics of RA, therefore, this review discusses the lipid level of RA patients, the relationship between disease progression and lipid level, and the effect of anti-rheumatic drugs or dietary intervention on the lipid mass spectrum of RA patients. We hope this review can deepen the understanding of RA from the perspective of lipid metabolism by providing updated evidence.

## 2 Changes in lipid metabolism in RA

### 2.1 Blood lipid profile of RA patients

Blood lipids refer to TG, cholesterol, and lipids (such as phospholipids and glycolipids) in plasma. Plasma proteins generally include apolipoproteins in blood and molecular complexes composed of a variety of lipids. In clinical practice, the parameters generally concerned with measuring blood lipid metabolism in RA patients are TG, total cholesterol (TC), low-density lipoprotein cholesterol (LDL-C), high-density lipoprotein cholesterol (HDL-C), phospholipids, FAs, lipoproteins, and apolipoproteins, et al. Previously, it was generally believed that the TC, LDL-C, and HDL-C of untreated patients with RA before and during the active period were reduced (15–17). Consistent with this, recent studies also found that HDL-C (16, 18–20) and LDL-C (17) decreased, and TC/HDL-C (16, 18) increased in RA patients. Studies on TC have both down-regulated results consistent with the previous report (19), and conflicting views, such as the level of TC in RA patients remained unchanged (18), or the level was up-regulated (20, 21). The serum lipoprotein (a) (Lp(a)) concentration of RA patients increased significantly (16, 19), and the apolipoprotein B (apo B)/apolipoprotein A-I (apo A-I) ratio was also significantly higher than that of the control group (16). In addition, the levels of circulating FAs in RA patients have also been detected. Compared with the control group, the percentage or concentration of palmitic acid, palmitoleic acid, oleic acid, linoleic acid, erucic acid, AA, EPA, 20-22C monounsaturated fatty acid (MUFA), and/or total n-6 PUFA decreased (22). Lipid derivatives, such as leukotriene B4 (LTB4) derived from AA, are elevated in RA due to their pro-inflammatory effects (23). About SPMs, resolvin D3 (RvD3), RvD4 and resolvin E3 (RvE3) decreased in the serum of RA patients (24). Although a single detection of plasma lipid profiles may display lipid levels in RA patients to some extent, there are always conflicting results in clinical detection due to differences in detection populations or methods. If new discoveries are to be made from this perspective, it is necessary to link lipid levels more widely with disease markers or related complications such as CVD, which will be beneficial for the clinical diagnosis and treatment of RA.

Actually, a few studies have begun to explore the relationship between plasm lipid change and inflammation related markers in RA patients (20). The level and duration of C-reactive protein (CRP) are closely related to the severity and activity of inflammation, so CRP is often used as an inflammatory marker of RA (25). In addition, erythrocyte sedimentation rate (ESR) is also commonly used to diagnose the degree of RA inflammation. Clinical diagnosis of RA should consider the influence of concentration of plasma lipids to make a reasonable judgment on the diagnosis of the disease. Compared with the control group, the average level of inflammation marker high-sensitivity CRP (hsCRP) in RA patients was higher. HDL-C was negatively correlated with hsCRP (20, 26), and higher TC/HDL-C and higher LDL/HDL ratios positively correlate with hsCRP (20). Furthermore, in patients in the increased inflammation cohort, the increase in hsCRP levels was associated with a significant decrease in LDL-C, TG, TC, apoB, and apoA-I levels (27). ESR and RA also showed gender correlation. ESR level was negatively correlated with HDL-C concentration in RA patients, and in male RA patients, ESR concentration increased with the increase of LDL-C (26). These results showed that the lipid levels exhibited a correlation with hsCRP and ESR to some extent.

To further understand the change of serum lipids in RA, different RA animal models were established for related research. Compared with clinical specimens, animal models can dynamically simulate the occurrence and progression of RA and provide theoretical support for clinical data. In the rat model of pristane-induced arthritis, it was found that the serum TC and TG were low in the acute phase and chronic phase after induction. In addition, adiponectin levels were low in the acute phase, but not in the chronic phase (28). In a more comprehensive collagen-induced arthritis (CIA) model, joint tissue and plasma samples were taken as research objects to study the possible ways of lipid changes in RA. Compared with the control group, TG, TC, and phospholipids in plasma lipids of CIA group rats decreased significantly, TC and phospholipids in joint tissue increased significantly, while TG slightly decreased without significant difference. TG, phospholipids, and TC in the joints of CIA rats were negatively correlated with TG, phospholipids, and TC in plasma (29). Moreover, the levels of AA and its metabolites, phospholipid metabolites, and EPA metabolites in the RA rat model were significantly increased, such as PGE2, PGF2α, PGI2, LTB4, and thromboxane B2 (30–32). There are also differences in the levels of oxylipins between CIA mice and healthy mice. Among them, the oxylipins derived from AA, DHA, and EPA were up-regulated, while the oxylipins derived from linoleic acid were down-regulated (33).

### 2.2 Lipid levels in synovial fluid of RA

Synovial fluid (SF) provides nutrition and lubrication for articular cartilage. Normal human SF contains very low concentrations of lipoproteins and apolipoproteins, and there is a significant difference between them and the lipid content in blood (34). In RA, SF accumulates at the synovial junction of the joint (35). SF of RA is rich in inflammatory cytokines and immune cells, which can further enhance synovial inflammation. Therefore, the detection results of SF in RA patients are usually compared with other bone diseases, such as osteoarthritis (OA). In terms of FA content, the percentage of palmitic acid, total saturated FA, long chain MUFA and/or total MUFA increased in SF of RA compared with SF of trauma control or OA patients (36). The myristic acid and palmitoleic acid levels were lower than those in patients with non-RA inflammatory arthritis (37). Interestingly, the lipid level in SF of RA was different in location (38). The proportion of AA and DHA in the SF of RA shoulder increased, and the proportion of oleic acid decreased. The proportion of linoleic acid, DHA, and total n-6 PUFAs in SF of the RA knee joint was low. These changes may affect joint lubrication, synovitis, pannus formation, cartilage, and bone degeneration, and lead to the pathogenesis of inflammatory joint disease (30). In terms of the content of SPMs, maresins 1 ll, 5S-hydroxy eicosapentaenoic acid (HETE), 12S-diHETE, RvD1, RvD3, RvD5, and lipoxins A4 (LXA4) were detected in SF of RA (39–41), and it was confirmed that the levels of PGE 2, 15-HETE and LTB4 increased in SF of RA (40, 42). Although there are currently few comparative studies on lipids in the joint synovial fluid between RA and normal individuals, some oxidized lipids have specificity in RA compared to OA patients, such as LXA4, 5-HETE, 12-HETE, etc. These specific oxidized lipids may serve as biomarkers for diagnosis.

## 3 Effect of lipids on RA

### 3.1 Effect of lipids on inflammation of RA

The pathophysiology of RA inflammation involves numerous cell types, including T cells, B cells, dendritic cells (DCs), neutrophils, macrophages, and fibroblast-like synoviocytes (FLS). Lipid metabolites can affect the progress of RA by regulating the activity and function of these cells (**Table 1**).

**Table 1:** Effect of lipid on Inflammation of RA.

Cell type	Lipid	Effects	Ref.
T cell	FA	Make T cells invasive and migratory	(43)
	AA	Increase the expression of ORAI3 of CD4^+^T cells and the sensitivity of T cells to AA	(10)
	Derivatives of cholesterol and FA	Cause imbalance of IL-17 expression through ROR γ T-IL-17 axis	(44)
	PGE2	Increase the expression of IL-17A, CD80 and CD86 in γδT cells	(45)
	N-3 PUFA MaR1	Stimulate the differentiation of Treg cells and regulate the balance of Treg/Th17	(46, 47)
B cell	Saturated FA	Regulate the differentiation of B cells through FFA2 receptors to alleviate RA	(48)
DC	PGE2	Regulate the production of IL-23 by DCs, leading to the expansion of Th17 cells	(49)
neutrophil	LTB4	Promote inflammation by participating in neutrophil anti-apoptosis and neutrophil infiltration	(50, 51)
FLS	Free FA	Increase the secretion of IL-6, IL-8, MCP-1, pro-MMP1 and MMP3	(52)
	15S-HETE	Increase the expression of MMP2 Enhance the production of placenta growth factor through PI3K-Akt, NF-κ B, and COX-2 pathways	(53, 54)
	LTB4	Induce FLS to produce TNF-α and IL-1β	(23, 55)
	PGE2	Increase the proliferation of FLS	(56)
	Adiponectin	Induce the expression of COX-2 and mPGES-1, increasing the production of PGE2 in FLS of RA	(57)
	LXA4	Induce the expression of COX-2 and membrane-associated PGE synthase-1, enhancing the production of PGE2 in FLS of RA	(58)
		Inhibit the proliferation of synovial cells, reduce the level of TNF-α, IL-6, IL-1β, and IFN-γ, inhibit p38 MAPK signaling pathway	(59)

#### 3.1.1 Effect of lipids on the immune cell of RA

##### 3.1.1.1 Effect of lipid on T cells and B cells of RA

As an autoimmune disease, RA is associated with immune cell dysfunction at different developmental stages. In RA, lipid abnormalities in T cells contribute to tissue invasion and migration, promoting synovial inflammation and eroding cartilage and bones (60, 61). At present, it is believed that lipid abnormalities in T cells are mainly caused by mitochondrial metabolic defects (60, 62, 63). Mitochondrial metabolic defects in T cells of RA are not conducive to glycolysis, thus shunting to pentose phosphate pathway, producing NADPH and biosynthetic precursors, and finally producing FAs. T cells adapt to the formation of excessive FAs by depositing lipid droplets in the cytoplasm. Subsequently, the T cell membrane containing lipid droplets was then arranged into invasive folds, which promoted the migration of T cells in the extracellular matrix and tissues (43).

Regarding other aspects of lipids and RA T cells, AA-regulated calcium signals in T cells of RA promote the development of synovitis. The expression of ORAI3, the pore-forming calcium channel component of CD4^+^T cells from RA patients, increased, thus enhancing the activity of AA-regulated calcium selective channels, making T cells sensitive to AA (10). Retinoid-related orphan nuclear receptor γt (RORγt) is expressed in mouse and human T cells, RORγt is the main transcription factor of interleukin (IL) -17, and the derivatives of cholesterol and FAs are also the natural ligand of RORγt (64). The imbalance of IL-17 expression is closely related to autoimmune diseases and inflammatory diseases, therefore RORγt-IL-17 axis is an interesting target for studying T cell function in RA (44). The number of γδT cells and the level of IL-17A increased in RA patients. In addition, after being treated with PGE2, the level of IL-17A in γδT cells increased correspondingly, and the expression of costimulatory molecules CD80 and CD86 in these cells also increased (45). Th17 and Treg subset dysregulations were present in RA patients (65). Fat-1 transgenic mice expressed the fat-1 gene, which encodes an n-3 FA desaturase that can convert n-6 FAs into n-3 FAs, producing abundant n-3 FAs that do not require dietary supply. This allows for the exploration of the role of n-3 PUFA by a collagen antibody-induced arthritis model. It found that n-3 PUFA decreased the expression of IL-6, IL-23, and IL-17, and stimulated the expression of FoxP3, thereby stimulating the differentiation of Treg cells (46). In the CIA model, MaR1 increased the proportion of Treg cells and decreased the proportion of Th17 cells, regulating the balance of Treg/Th17, thereby improving RA progress (47).

Compared with T cells, the effect of lipids on B cells in RA is less studied at present. Although B cells participate in the disease process of RA through antigen presentation, cytokine secretion, and autoantibody production, not all B cells can promote the pathogenesis of RA, and some antibodies produced by B cells can prevent and protect RA (66). It is reported that short-chain FAs regulated the differentiation of B cells through its receptor FFA2 to alleviate RA (48).

##### 3.1.1.2 Effect of lipids on dendritic cells, neutrophils, and macrophages of RA

In addition to adaptive immune cells, some innate immune cells including DCs, neutrophils, and macrophages also play important roles in the progression of RA. In DCs, in addition to the role of energy storage and structural components of the cell membrane, lipids act as second messengers and effectors in the steps of cell differentiation and regulate important functions of DCs (67). The chronic inflammatory environment in RA seriously affects the distribution and function of DCs, resulting in tolerance deficiency and aggravation of inflammation. The synovial fluid of RA patients is rich in DCs subpopulations derived from monocytes, which can promote the harmful Th17 response (68). Synovial cells release PGE2 to produce IL-23, IL-6, IL-1β, and TNF-α through the EP2/EP4 (membrane receptors) signal of DCs, which leads T cells in arthritis to differentiate into Th17 cells. Subsequently, Th17 induces the production of IL-1β, IL-6, matrix metallopeptidase-1 (MMP-1), and MMP-3 through synovial cells, further promoting the release of PGE2 in synovial cells. This process establishes a pro-inflammatory cycle in RA through DCs (49).

Neutrophils account for over 90% of the cells found in the synovial fluid of RA patients. Synovial fluid contains various immune complexes and complement components. Previous studies have shown that inflammatory mediators (PGE2, TXA2, LTB4) released by neutrophils after ingesting immune complexes and complement components in synovial fluid are one of the causes of RA inflammation. These inflammatory mediators can inhibit the functions of neutrophils, platelets, macrophages, and mast cells. As a product of AA, LTB4 is considered the most potent proinflammatory agent (69, 70). LTB4 promotes inflammation in RA by participating in neutrophil anti-apoptosis and neutrophil infiltration (50, 51).

Lipids and macrophages in inflammation are extensively studied. On the one hand, lipids serve as membrane components, and the lipid composition of cell membranes can alter membrane fluidity or lipid raft structure, affecting cellular signaling pathways (71). Changes in cholesterol content and lipid raft composition reduce macrophage inflammation in the absence of FA synthase (72). On the other hand, exogenous lipids, long-chain saturated FAs, such as palmitate or stearate, can act directly as pro-inflammatory signals (73). Derivatives of certain FAs, such as PGs, thromboxanes, leukotrienes, resolvins, and maresins, can act as immunomodulators, mediating the effects of macrophages (74). Although there has been extensive research on the role of macrophages with lipids in inflammation, in RA, apart from the metabolic features of lipid-scavenging macrophages that will be described in the 3.3 section, there are currently few studies that combine the immune effects of macrophages with lipids, and there is still a long way to go in the future.

#### 3.1.2 Effect of lipid on fibroblast-like synoviocytes of RA

Synovitis is the pathological basis of RA. In RA, FLS, also known as synovial fibroblast, is the most common cell type at the pannus cartilage junction. It invades and degrades the cartilage matrix by producing cytokines, chemokines, and matrix degradation molecules, leading to joint destruction (75). Compared with FLS of OA, the levels of 12-22C saturated FA, palmitoleic acid, oleic acid, and linoleic acid in FLS of RA are higher (76).

In RA, lipids affect FLS function in many ways. Free FA contributes to the pro-inflammatory environment of FLS in RA. In FLS, free FA dose-dependently increases the secretion of pro-inflammatory cytokines, chemokines, and matrix degrading enzymes (such as IL-6, IL-8, monocyte chemoattractant protein-1 (MCP-1), pro-MMP1 and MMP3). And the effects of saturated and unsaturated free FA on FLS in promoting inflammation are similar (52). TLR4 is involved in FA-induced signal transduction (77). Palmitic acid can induce the secretion of IL-6 in FLS. However, after the use of inhibitors to inhibit extracellular and intracellular TLR4, PA-induced IL-6 secretion was inhibited through FA transporter CD36/FA translocase (52). Bioactive lipids have various regulatory effects on FLS. A kind of AA metabolite, the downstream product of 15-LOX of 15S-HETE, can increase the mRNA and protein levels of MMP2 in FLS from RA patients (53). 15S-HETE can also enhance the production of placenta growth factor in FLS through PI3K-Akt, nuclear factor κ-B (NF-κ B), and cyclooxygenase-2 (COX-2) pathways (54). Another AA metabolite, LTB4, is an effective pro-inflammatory lipid mediator. Its expression in active RA patients is higher than that in inactive RA patients and healthy donors. The main LTB4 receptor expressed on FLS is LTB4 receptor 2. LTB4 induces FLS of RA to produce TNF-α and IL-1β through LTB4 receptor 2 (23, 55). PGs are beneficial to leukocyte infiltration, synovial hyperplasia, and angiogenesis, thus promoting synovitis (78). Compared with OA and joint trauma patients, PGE2 and its processing enzymes COX-2 and membrane-associated PGE synthase-1 have the highest content in synovial tissue of RA patients, mainly in FLS and plasma cells (79). FLS promotes the expression of triggering receptor expressed on myeloid cells-1 in monocytes through the COX-2/PGE2 pathway (80). Adiponectin induced the expression of COX-2 and membrane-associated PGE synthase-1, thereby enhancing the production of PGE2 in the FLS of RA (57). Other bioactive lipids may protect FLS through their anti-inflammatory effects. For example, 15-LOX induced by IL-13 may regulate the production of LXA4, thus having an anti-inflammatory effect (81). *In vitro* experiments confirmed that LXA4 could inhibit IL-1β induced production of IL-6, IL-8, and MMP3 in human FLS, and enhance the synthesis of tissue inhibitors of metalloproteinases (58). *In vivo* experiments demonstrated that LXA4 could inhibit the proliferation of synovial cells of CIA mice, reduce the level of TNF-α, IL-6, IL-1β, and IFN-γ, and inhibit the p38 MAPK signaling pathway (59).

### 3.2 Effect of lipids on cartilage and bone of RA

Lipids play an important role in bone remodeling in the microenvironment of bone. On the one hand, the interaction between osteoblasts and cholesterol contributes to bone homeostasis, and adipogenic molecules contribute to the differentiation balance of osteoblasts. On the other hand, osteoclasts can respond to changes in metabolic dysfunction (82). RA patients often suffer from progressive injury of joint bone and cartilage, bone loss around joints and the whole body increases the risk of fracture in RA patients. Bone loss in RA patients is the result of excessive osteoclasts (OCs) mediated absorption and limited osteoblasts (OBs) mediated formation (83). OBs are the main functional cells of bone formation, responsible for the synthesis, secretion, and mineralization of bone matrix. On the contrary, OCs mediate bone resorption during bone transformation and OCs over-absorb bone in RA (84). Cartilage is the key component of the synovial joint. In addition to various matrix proteins, chondrocytes are the only cells in cartilage. There are many inflammatory mediators in the synovial joints of RA patients. Chondrocytes can not only act as target cells of these inflammatory mediators but also lead to cell dysfunction. Chondrocytes can also act as effector cells to directly or indirectly promote joint injury of RA (85). The role of lipid regulation in the bone cells and chondrocytes of RA is complex (**Table 2**).

**Table 2:** Effect of lipid on bone cells and chondrocytes of RA.

Cell type	Lipid	Effects	Ref.
Osteoclast	FA	Induce the fusion of OCPs and promote the destruction of RA joints	(86)
	PA, LA	Increase the secretion of IL-8	(87)
	LeukotrieneB4	Stimulate differentiation of OCs by increasing RANKL expression of FLS Regulate OCs differentiation negatively through NF-κ B and Ca^2+^signals	(88, 89)
	LPA1	Promote the development of arthritis through OC generation and cell infiltration	(90)
	DHA	Suppress c-Jun N-terminal kinase, ERK, and p38/mitogen-activated protein kinase pathway Suppress NF-κ B-signal cascade, weakening the induction of c-Fos and nuclear factor of activated T cells c1 Induce the expression of Bim to accelerate the apoptosis of mature OCs Inhibit the proliferation and differentiation of bone marrow-derived macrophages and promote the apoptosis of OCs	(91)
	RvE1	Inhibit the expression of nuclear factor of activated T cells c1 and c-fos in OCs, inhibiting the formation of OC and bone resorption	(92)
Osteoblast	PA, LA	Increase the secretion of IL-6, IL-8, growth-related oncogenes α and MCP-1	(87)
chondrocyte	PA	Induce the production of IL-6	(52)
	PGE2	Activate protein kinase A and protein kinase C signals to slow down the maturation of chondrocytes	(93)
	17R-RvD1	Reduce the biosynthesis of PGE2 and increase the level of protective SPMs	(39)
	RvD1	Reduce cartilage erosion by miRNA-146a-5p	(94)

#### 3.2.1 Effect of lipid on osteoclasts of RA

OCs are derived from monocytes/macrophage precursors and developed into OCs after the activation of macrophage colony stimulating factor and receptor activator of nuclear factor-κB receptor ligand (RANKL) (95). Among them, the RANKL-RANK signal plays a key role in the generation and function of OCs (96). TNF receptor-associated factor 6 activated by RANKL signal transduction leads to the activation of NF-κ B and mitogen-activated protein kinase (MAPK), including c-Jun N-terminal kinase and p38 (97). Through the examination of the bone erosion site at the bone pannus interface of RA patients, multinuclear giant cells could be found, which were tartrate-resistant acid phosphatase positive, calcitonin receptor-positive, and cathepsin K positive (98).

Osteoclast precursors (OCPs) show monocyte phenotype after being stimulated by macrophage colony stimulating factor and RANKL, and circulating OCPs play a crucial role in bone erosion of RA. However, increasing FA oxidation can induce OCPs fusion and promote RA joint destruction (86). The differentiation of OCs induced by free FA, PA, and linoleic acid enhanced the secretion of IL-8 compared with OA, especially at the earliest point of differentiation, indicating that the sensitivity of RA to free FA increased (87). LTB4 can promote OCs formation and induce bone injury in many ways. On the one hand, LTB4 can indirectly stimulate human OCs differentiation by increasing RANKL expression of FLS (88). On the other hand, as a receptor of LTB4, BLT1 regulates OCs differentiation negatively through NF-κ B and Ca^2+^ signals, thus linking the dual role of the LTB4 receptor in OCs formation (89). Lysophosphatidic acid is a bioactive lipid that can bind to LPA receptors. LPA1 is highly expressed in the synovium of RA patients, and LPA/LPA1 signal promotes the development of arthritis through OCs generation and cell infiltration (90).

Although some lipids and their derivatives can promote bone resorption of OCs, other lipids can inhibit OCs formation by promoting apoptosis or other signaling pathways, thus playing a role in bone protection in RA. DHA inhibited c-Jun N-terminal kinase, ERK, and p38 MAPK (91). In addition, DHA can inhibit NF-κ B signal cascade, which weakens the induction of c-Fos and nuclear factor of activated T cells c1 (91). DHA plays an anti-osteoclastogenic role by inhibiting the proliferation and differentiation of bone marrow-derived macrophages and enhancing the apoptosis of mature OCs (91). DHA accelerates the apoptosis of mature OCs by inducing the expression of Bim, which is a key regulator of OCs apoptosis (91). RvE1 inhibits the expression of nuclear factor of activated T cells c1 and c-fos in OCs induced by RANKL, thus inhibiting the formation of OCs and bone resorption (92).

#### 3.2.2 Effect of lipid on osteoblasts of RA

Although OBs play an important role in RA bone loss, the effect of lipid metabolism on OBs is less concerning. Current research mainly focuses on free FA. The effect of free FA on OBs of RA is multifaceted (87). When stimulated with free FA, such as PA or LA, OBs of RA secretes more pro-inflammatory cytokines IL-6, chemokine IL-8, and growth-related oncogenes α and MCP-1 (87). The mineralization activity of OBs is negatively correlated with the level of IL-6 secretion induced by free FA (87). TLR4 is considered the receptor of free FA and is involved in arthritis-dependent joint destruction. After specifically blocking the expression of LTR4, the IL-8 secreted by PA-induced OBs decreased (87).

#### 3.2.3 Effect of lipid on chondrocytes of RA

In addition to bone destruction, cartilage damage is also a typical feature of RA. The stimulation of PA induced human chondrocytes to secrete a large amount of IL-6. However, the stimulation of unsaturated FAs, especially PUFAs, has a weak effect on the secretion of IL-6 by human chondrocytes (52). PGE2 is a regulator of chondrocyte maturation rate, which can activate the response reporter genes in cAMP response element/cyclic AMP response element binding protein and AP-1, and play a role through protein kinase A and protein kinase C signaling pathways. The activation of the cAMP response element/cyclic AMP response element binding protein signal of PGE2 could be completely blocked by inhibition of protein kinase A/cyclic AMP response element binding protein and partially blocked by inhibition of c-Fos/PKC, thus suppressing chondrocyte differentiation (93). RvD1 often shows cartilage protection in RA. RvD1 can significantly reduce the serum marker of cartilage renewal (99). The stable epimer 17R-RvD1 can prevent inflammatory arthritis in mice by significantly reducing the biosynthesis of PGE2 and increasing the level of protective SPMs (39). RvD1 can also reduce the cartilage erosion of CIA mice through miRNA-146a-5p (94).

### 3.3 Effect of lipid on CVD in RA

In addition to systemic inflammation and joint destruction, RA has other extraarticular manifestations, of which CVD is the most serious (100, 101). Lipid abnormalities help accelerate atherosclerosis and increase the risk of CVD, which is the main cause of increased mortality in RA patients (102). Although the lipoprotein level of RA patients is lower than that of the general population, the risk of death caused by CVD is significantly increased, which is known as the “lipid paradox”. Patients with RA had elevated TC, LDL-C, LDL/HDL-C, TC/HDL-C ratio, and decreased HDL-C (103, 104). CVD risk in RA patients is associated with elevated levels of LDL-C, TG, and hsCRP (105). In the follow-up cases of RA, it was found that the CVD incidence rate of male RA patients was higher than that of female patients because the TC of male patients was higher than that of female patients (8). A retrospective study of RA patients in China found that the incidence rate of CVD was significantly higher than those of patients with HDL ≤ 1.04 mmol/L (106). In RA patients, myeloperoxidase can mediate an increase in HDL oxidation, and it is more prominent in patients with CVD (107). Paraoxonase 1 (PON1) can metabolize oxidized lipids and participate in maintaining oxidative balance, and the activity of PON1 is lower in RA patients (108). Compared with the healthy control group, the myeloperoxidase/PON1 ratio of RA patients with a history of CVD was higher, indicating that HDL dysfunction determined by myeloperoxidase/PON1 ratio may be related to the increase of CVD in RA (109).

The increase of LDL level is considered as a strong predictor of CVD, the more LDL-C is reduced by drugs, the greater the subsequent reduction of CVD risk (105). In RA, compared with the control group, the level of small dense LDL granules was higher, while the level of small HDL granules was lower (110). Because small dense LDL particles are easier to penetrate endothelial cells than their larger counterparts and are more susceptible to oxidation (111). LDL transports cholesterol from the liver to tissues, and excessive accumulation of cholesterol in tissues will lead to atherosclerosis. When the endothelial cells of the artery wall change, LDL enters, and the small dense LDL is converted into oxidized LDL, which is eventually swallowed by macrophages to form foam cells through innate and adaptive immunity, causing plaque lesions (111–113). L5 is the most negatively charged subcomponent of LDL, which is related to atherosclerosis. In RA, L5 may promote atherosclerosis by enhancing the formation of macrophage foam cells, up-regulating the expression of M1 macrophage-related markers in vascular plaque formation and atherosclerosis, and improving the expression level of atherosclerosis-related mediators (such as IL-6, IL-8 and TNF-α) (111). RA plasma adversely affects the ability of monocytes/macrophages in the arterial wall to metabolize cholesterol and maintain lipid homeostasis, leading to premature atherosclerosis (114). Lp(a) is rich in cholesterol and consists of LDL particles attached to apoA. The increase of serum Lp(a) is related to the increased risk of atherosclerosis. As a positive acute phase protein, the mechanism of Lp(a) increase during inflammation may be due to the increased synthesis and up-regulation of Lp(a) expression (115).

In healthy individuals, in the absence of oxidative stress and systemic inflammation, HDL has an anti-inflammatory effect due to its function of reverse cholesterol transport and thus has cardioprotective properties (116). While in RA patients characterized by oxidative stress and systemic inflammation, the normal protective anti-inflammatory HDL changes to pro-inflammatory HDL due to changes in their structure and enzymatic content. During the acute phase reaction, serum amyloid A, apolipoprotein J, and pancreatic phospholipase A2 are present in the serum at high concentrations and mixed with HDL to replace their common components, such as cholesteryl ester transfer protein. Cholesteryl ester transfer protein enzyme transfers cholesterol esters to lipoproteins containing apo B, which are then absorbed by the liver to assist HDL to transport cholesterol to the liver and be absorbed. In addition, PON1 decreased and platelet-activating factor acetylhydrolase levels increased in HDL particles. PON1 has anti-inflammatory properties while platelet-activating factor acetylhydrolase has pro-inflammatory properties, these changes make HDL have the characteristics of promoting atherosclerosis (111, 117).

## 4 Strategies for regulating abnormal lipid metabolism in RA

### 4.1 Drugs affecting the blood lipid profile of RA

Common RA drugs include nonsteroidal anti-inflammatory drugs, statins, glucocorticoids, traditional disease-modifying antirheumatic drugs (DMARDs), and biological agents. Because of the different targets of pathophysiological pathways in the treatment of RA, they have different effects on the remission of RA. For example, nonsteroidal anti-inflammatory drugs are used to control the symptoms of RA. The therapeutic effect of statins is mainly attributed to their anti-inflammatory and immunomodulatory properties (118). In addition, statins can reduce the risk of CVD in RA patients through a lipid-lowering effect (119, 120). We will mainly review the effects of traditional DMARDs and biological agents on lipid and lipoprotein parameters in RA patients.

#### 4.1.1 DMARDs

Methotrexate (MTX) is the commonly used DMARDs in RA. The changes in plasma metabolome in patients with RA before and after MTX treatment were evaluated by semi-targeted metabolomics. It was found that the changes in multiple metabolites were related to the efficacy of MTX, including TC, FAs, and metabolites related to FA/lipid and energy metabolism (121, 122). As anti-atherosclerotic proteins, ATP binding cassette transporter-A1 and 27 hydroxylase are known to promote cell cholesterol efflux. Compared with other subjects taking DMARDs, there was no difference in blood lipid levels in patients taking MTX, while the expressions of ATP binding cassette transporter-A1 and 27 hydroxylase were significantly increased (121). However, using serum lipidomics to predict the response of RA patients to MTX, it was found that the detection of serum lipid profile before or at the early stage of treatment has no supporting effect in routine clinical practice (123). As an antimalarial drug, hydroxychloroquine (HCQ) has the characteristics of DMARDs and is commonly used in the treatment of mild RA (121). It can improve the blood lipid level of RA patients. Patients taking HCQ showed significantly lower TC, LDL, TC/HDL, and LDL/HDL and higher HDL (124, 125). Consistent with these results, the oral administration of 400mg/day HCQ within three months reduced the levels of serum TC and TG in a clinical trial of 15 RA patients (126).

#### 4.1.2 Biological agents

Anti-TNF-α agents and anti-IL-6 agents are common biological agents for the treatment of RA, which can effectively alleviate inflammation (127). TNF-α, a key inflammatory cytokine, can induce atherogenic changes in lipid mass spectrometry and may increase the cardiovascular risk of RA patients. The use of anti-TNF-α agents is related to the changes in serum lipid profile in RA patients, but some research results are contradictory. At present, the common biological agents used to treat RA are infliximab (IFX), etanercept (ETN), adalimumab, certolizumab pegol, and golimumab (128). Whether compared with baseline or non-TNF-α inhibitor users, TNF-α inhibitors were not related to the difference in blood lipid level in RA patients, including TC, TG, LDL-C, HDL-C, LDL/HDL, TC/HDL, apo A-I, apo B and apo B/apo A-I (129, 130). Another study found no change in the levels of TC, HDL-C, LDL-C, and atherogenic index, while TG levels increased significantly (131). ETN treatment resulted in a significant and sustained decrease in the apo B/apo A-I ratio in patients with good or moderate EULAR response (132). Neutralizing TNF with monoclonal anti-TNF antibody (one kind of adalimumab) increased HDL-C levels and decreased CRP and IL-6 levels after 2 weeks (133). The effect of IFX treatment on blood lipid levels seems to be neutral because there is no significant change in LDL-C level and TC/HDL-C and TG/HDL-C ratio during treatment (134). Another similar study suggests that IFX can change the blood lipid level of patients with active RA, TC, TG, HDL-C, LDL-C, and apoB levels increased significantly from baseline, while ATI, LDL-C/HDL-C ratio, and LDL-C/apoB ratio remained unchanged (135). In patients who successfully received IFX or MTX treatment, although the serum TC level increased and normalized, there was a difference in the regulation of lipid components between IFX and MTX, for example, IFX treatment preferentially induced ultra-high levels of VLDL-TG. Therefore, it is necessary to pay attention to the level of TG during IFX treatment (136). In addition, IFX was beneficial to lipids by changing the antioxidant capacity of HDL and contributing to the protective effect of anti-TNF agents on CVD in RA (137). However, some studies believed that the beneficial effect of anti-TNF-α treatment on CVD was not related to the effect on lipid metabolism (129, 134). TNF-α blocking therapy had no significant overall effect on the atherosclerosis index in RA patients (138, 139). JAK inhibitor (JAKi) can target and block cytokine signal transduction mediated by Janus kinase signal transduction and transcriptional activator pathway, thus regulating immune response and cell growth. Targeted inhibition of JAK in autoimmune diseases such as RA can effectively control diseases (140). A study reported that JAKi could reduce pain reported by RA patients. In JAKi treated patients, there is a significant association between pain reduction and DHA changes, and the analgesic effect may be related to n-3 FAs and increased DHA levels (141).

In short, studies on changes in lipid levels after drug treatment, to further identify potential metabolic biomarkers of response to drug treatment, or to combine lipid levels with other pathological symptoms accompanying RA, such as diabetes and CVD, can not only guide drug treatment of RA through lipid metabolism but also explore the therapeutic effects of drugs on complications such as CVD from multiple perspectives, such as metabolism and immunity.

### 4.2 Dietary intervention

In addition to drug treatment, diet also has beneficial or harmful effects on RA. Overall, energy intake, total FA and saturated FA, the unbalanced ratio of n-3 to n-6 FA, and the western type diet characterized by a high sugar diet, low fiber, and low antioxidant content can increase obesity, thereby increasing the risk of RA (142, 143). Therefore, dietary intervention strategy in RA is also a hot spot in RA research. The anti-inflammatory diet has a positive effect on the disease activity of RA (144). It has been found in different systematic reviews of randomized controlled trials that a Mediterranean diet can reduce the disease activity and drug treatment failure rate of RA. Although vitamin D supplementation does not affect disease activity score 28 (DAS28), it is beneficial to the outcome of RA (142, 145). 1.7-3 g/d of n-3 FA did not affect DAS28, but 3-6 g/d of n-3 FA could effectively reduce RA-related pain (145, 146). Accumulation of toxic lipid mediators in skeletal muscle can induce mitochondrial dysfunction and insulin resistance, which are key determinants of CVD and sarcopenia. Most clinical trials have shown that n-3 PUFA supplements are beneficial to RA patients (147–150). High n-3 PUFA intake can prevent the occurrence of RA (147, 151, 152) and improve the prognosis of RA (41, 148). N-3 FA can improve muscle metabolism and limit muscle atrophy in obese and insulin-resistant subjects, thereby preventing CVD, poor activity, and death risk in RA patients (153). In a longitudinal study, compared to the group with lower n-3 FAs intake, RA patients with high levels of n-3 FAs, palmitoleic acid, and AA in their diet showed a decrease in arterial stiffness, which helps to slow the progression of arterial aging (154). In addition, through the meta-analysis of RA disease severity markers, it was found that oral n-3 FA was beneficial to RA disease activity and improved the disease severity markers (155). Dietary regulation also has a certain effect on the SPMs level of RA patients. Marine oils are a rich source of n-3 FAs, especially the essential FAs, DHA, and EPA (156). By supplementing enriched marine oil supplements, the concentration of SPMs in the peripheral blood of RA patients increased and the peripheral blood cells were reprogrammed. SPMs have a guiding role in mediating the immune response of this supplement (157). After supplementation with n-3 PUFA, LTB4 levels were decreased in RA subjects (155, 158). In conclusion, a reasonable diet or dietary supplementation, such as a Mediterranean diet, supplemented with enriched marine oil supplements or n-3 FA, can suppress the clinical symptoms of RA to a certain extent. The current difficulty is that a broad and applicable dietary standard cannot be proposed, but in clinical treatment, doctors can provide reasonable dietary recommendations to assist patients.

Compared with only receiving RA drugs, proper dietary supplementation has an effective effect on delaying the progression of RA disease. Data from a population-based prospective study in Sweden found that a higher intake of dietary vitamin D and n-3 FA can enhance the therapeutic effect of DMARDs (159). Supplementation of n-3 FA with indomethacin may improve disease activity compared to only receiving indomethacin, or supplementation with n-3 FA (160). In the CIA mouse model, the combination of curcumin and n-3 FA delayed disease progression, reduced disease severity, and significantly inhibited the factors related to RA occurrence, such as TNF, IFN-γ, and MCP-1 (161). Long-term combined supplementation of coenzyme Q10 and n-3 FA can restore the damaged mitochondrial bioenergy and antioxidant status in RA animal models (162). Higher plasma EPA is associated with a greater reduction of DAS28, and EPA is associated with clinical improvement of anti-TNF therapy *in vivo*. ETN increases Th17 frequency *in vitro*, and EPA prevents the effect of ETN on Th17 cells (163). Clinically, in addition to exploring the therapeutic effect of drugs on RA, it is also important to explore the early intervention of diet in RA progression or the therapeutic effect of synergistic drugs. There is a long way to go for systematic drug therapy and food therapy.

## 5 Conclusion and perspectives

Although RA is still incurable, the development of DMARDs and the improvement of treatment schemes make it an almost controllable disease. Like the pathological mechanism of RA, lipid metabolism in RA is also complex. At present, more and more attention has been paid to the relationship between lipid metabolism and the progress of RA and the changes in plasma lipid levels after different drug treatments, drug combinations, or dietary interventions. As the most abundant type of cellular metabolites, lipids play an undeniable role in energy supply and storage, cell membrane construction, transportation, and signal transduction. Secondly, using lipids and lipid mediators in plasma as diagnostic and therapeutic targets not only facilitates clinical detection but also enables more targeted treatment based on individual differences. However, there are still many shortcomings in related research. Firstly, there are often conflicting results regarding lipid levels due to differences in population, experimental duration, or analytical methods. Secondly, it is currently unclear whether changes in lipid metabolism are the cause, consequence, or both of RA. In addition, at the cellular immune level, some cells, such as B cells, neutrophils, and macrophages, have been extensively studied in lipid metabolism, but there is still a lack of relevant evidence in RA. Finally, although some lipids have the effect of promoting RA progression, there are still various types of lipids and their metabolites, such as DHA, EPA, and the oxylipins derived from them (resolvins, maresins, and protectins), which have been proven to have protective effects on RA. How to guide the treatment or intervention of RA from the perspective of lipids and provide corresponding explanations from a molecular perspective remains a challenging task. Therefore, we need further research to address existing issues to better determine the pathophysiology of lipid abnormalities and the potential impact of drug therapy on RA lipid metabolism. To better integrate the procession of lipid metabolism with the prevention, occurrence, development, and treatment of RA and even related complications will undoubtedly enable us to have a more comprehensive understanding of RA.

## Author contributions

QL wrote the manuscript. JY participated in the writing. LL, NZ, and CL helped in revising the manuscript. AL and XH conceptualized the idea and revised the manuscript. All authors read and approved the final manuscript.
